# Bovine herpes virus type 1 induces apoptosis through Fas-dependent and mitochondria-controlled manner in Madin-Darby bovine kidney cells

**DOI:** 10.1186/1743-422X-9-202

**Published:** 2012-09-17

**Authors:** Xingang Xu, Kuan Zhang, Yong Huang, Li Ding, Guangda Chen, Honglei Zhang, Dewen Tong

**Affiliations:** 1 College of Veterinary Medicine, Northwest A&F University, Yangling, Shaanxi, 712100, PR China

**Keywords:** BHV-1, MDBK cells, Apoptosis, Caspase cascades, Fas, Mitochondria

## Abstract

**Background:**

Bovine herpesvirus type 1 (BHV-1) is an important pathogen in cattle that is responsible for substantial economic losses. Previous studies suggest that BHV-1 may induce apoptosis in Madin-Darby bovine kidney (MDBK) cells via a mechanism only involving caspases and p53. However, the mechanism for BHV-1-induced MDBK cell apoptosis still requires more research.

**Methods:**

MDBK was used as a model to study the precise signaling pathways of apoptosis induced by BHV-1 infection.

**Results:**

BHV-1 infection activated a Fas/FasL-mediated apoptotic pathway, resulting in activation of caspase-8 and cleavage of Bid. In addition, BHV-1 infection down-regulated Bcl-2 and up-regulated Bax expression, thereby initiating the release of cytochrome c followed by caspase-9 activation. The combined activation of the extrinsic and intrinsic pathways resulted in activation of downstream effecter caspase-3 and poly ADP-ribose polymerase (PARP), leading to apoptosis. Furthermore, blocking apoptosis using caspase inhibitors improved BHV-1-infected MDBK cell viability to different extent. BHV-1 infection did not induce significant DNA fragmentation in MDBK cells pretreated with ammonium chloride (NH_4_Cl) or cells infected with UV-inactivated BHV-1. Blocking caspases activation increased BHV-1 replication.

**Conclusions:**

BHV-1 induces apoptosis in MDBK cells through extrinsic and intrinsic pathways and there might be cross-talk between the two pathways. In addition, BHV-1 replication may be necessary for the induction of apoptosis in BHV-1-infected cells, and prolonged cell viability benefits BHV-1 replication.

## Background

Bovine herpes virus type 1 (BHV-1), an alphaherpesvirinae subfamily member, is an important pathogen in cattle that gives rise to substantial economic losses as a result of effects including reproductive failures, increased calf mortality, as well as enteric and respiratory disease. As a viral pathogen in cattle, BHV-1 causes severe respiratory infection, conjunctivitis, abortion, vulvovaginitis, balanopostitis, and systemic infection in neonate calves [1]. Most of these problems are caused by increased susceptibility to secondary infection which correlates with BHV-1-induced immunosuppression [2,3]. This immunosuppression may be partly due to apoptosis of infected lymphocytes because reduction of CD4^+^ T lymphocytes was detected in peripheral blood mononuclear cells (PBMCs) and lymph nodes during acute infection of BHV-1 and those CD4^+^ T lymphocytes undergo apoptosis [4].

Apoptosis is a major form of death caused by some types of virus infection. This process is characterized by detachment, plasma membrane blebbing, nuclear collapse and chromatin condensation. An important regulatory event in the apoptotic process is the activation of caspases, a family of cysteine proteases. Caspase cascades are involved in both intrinsic and extrinsic signal pathways which regulate the apoptotic process [5]. The relationship between virus infection and apoptosis is bidirectional. On one hand, virus infected cells can be eliminated by apoptosis mediated by components in the innate and adaptive immune systems. On the other hand, viruses have evolved strategies to regulate apoptosis, either by blocking a specific step of apoptotic cascade within the host cells, and thus maximizing virus production, or by actively inducing apoptosis, which consequently facilitates the spreading of virus progeny [6].

A previous study found that BHV-1 could induce PBMC subpopulations (B lymphocytes, T lymphocytes and monocytes) to undergo apoptosis individually [7]. Moreover, although penetration of BHV-1 is not required, attachment of BHV-1 viral particles is essential for the induction of apoptosis [8]. Also, it has been proved that the apoptosis induced by BHV-1 infection in MDBK cells involves p53-dependent mechanism [9]. To better understand the apoptotic process initiated by BHV-1 infection, in this work we further characterize caspases activation cascades during BHV-1 induced apoptosis in MDBK cells, focusing on the cell surface death receptor pathway and the mitochondria-initiated pathway. Our results demonstrate that BHV-1 infection appears to activate caspase-8 by Fas-dependent mechanism and to turn on caspase-9 by the mitochondria-dependent pathway. In addition, the two pathways could be associated through Bid.

## Results

### BHV-1 infection induced apoptosis in MDBK cells

**Figure 1:**
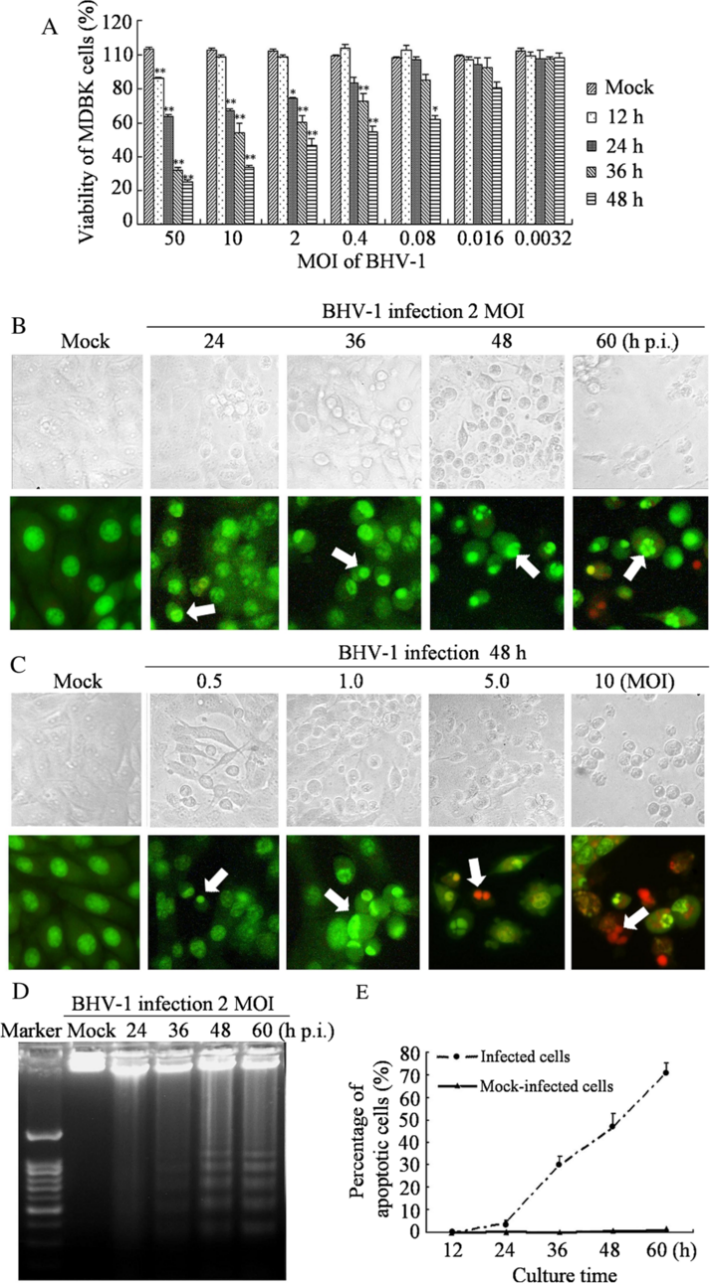
**Apoptosis induced by BHV-1 in MDBK cells.** (**A**) Cell viability changes of BHV-1-infected MDBK cells. Cells were seeded into 96-well culture palates and infected with BHV-1 at different MOI for indicated hours. Cell viability was detected by MTT assay. (**B**) Morphological changes of BHV-1-infected cells at 2 MOI for indicated hours. (**C**) Morphological changes of BHV-1-infected cells at different MOI for 48 h. Photomicrographs at inverted microscope of BHV-1-induced CPE (upper panel); Morphological changes under fluorescence microscopy followed by AO and EB staining (lower panel). Arrows indicate condensed chromatin and nuclear fragmentation. (**D**) DNA fragmentation of BHV-1-infected MDBK cells. Cells were mock-infected or infected with BHV-1 at 2 MOI for indicated hours, and then analyzed by DNA fragmentation assay. (**E**) Percentage of apoptotic cells. Cells were infected with 2 MOI BHV-1 at different time, and then detected by flow cytometry. Data are mean ± SD from three independent experiments. * *p < 0.05*, ** *p* < 0.01 versus mock infection.

To determine the susceptibility of cells to BHV-1 in our experimental system, cultured MDBK cells were infected with BHV-1 and then cell viability and the morphological changes in BHV-1-infected MDBK cells were determined. Infection of MDBK cells with BHV-1 resulted in cell death in a time-dependent manner, as detected by MTT assay. The loss of viability was also dependent on the MOI at which the cultures were infected (Figure 1A). Infection with amounts as low as 0.0032 MOI of BHV-1 had almost no impact on cell viability, however, with 50 MOI of BHV-1 only about 20% of the cells were alive at 48 h p.i. The reduction in cell viability at 2 MOI appeared about 24 h p.i., which became more evident at 36 and 48 h p.i. compared with mock-infected cells. Cells that were infected with 2 MOI of BHV-1 for indicated hours (Figure 1B, upper panel), or with different MOI for 48 h (Figure 1C, upper panel) were observed by an inverted microscope. Cytopathic effects (CPE) in the infected cells increased in a time-dependent and MOI-dependent manner.

Consistent with the CPE resulting from BHV-1 infection, mock-infected and BHV-1-infected MDBK cells were examined for characteristic signs of apoptosis using fluorescent microscope, agarose gel electrophoresis and flow cytometry. The BHV-1-infected cells showed typical apoptotic features with chromatin condensation and nuclear fragmentation to different extent after AO/EB staining (Figure 1B and C, lower panel). As shown in Figure 1D, DNA ladders could be detected as early as 24 h p.i., as MDBK cells were infected with BHV-1 at 2 MOI. Flow cytometry was used to measure the percentage of apoptotic cells. Increased rates of apoptotic cells were detected in BHV-1-infected MDBK cells. The percentage of apoptotic cells increased from 4.1% at 24 h p.i. to 70.5% at 60 h p.i. (Figure 1E). In contrast, mock-infected MDBK cells at corresponding times did not show obvious apoptosis. The above results provided biochemical evidence that the cell viability reduction and morphological changes observed in the BHV-1-infected MDBK cells are due to the induction of apoptosis.

### BHV-1 infection provoked activation of caspase-8, 9 and 3

**Figure 2:**
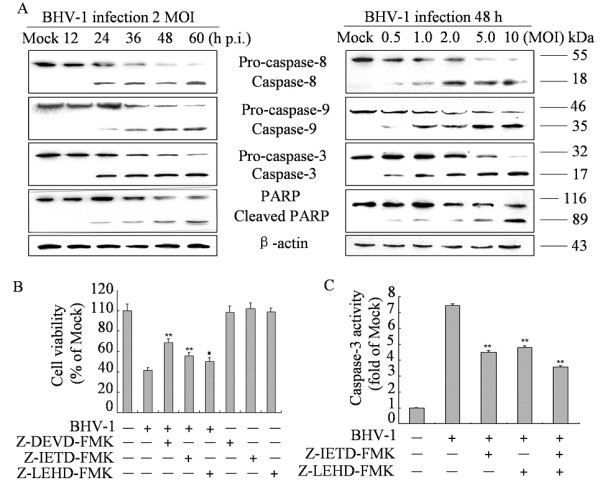
**Effects of BHV-1 infection on caspases and PARP cleavage in MDBK cells.** (**A**) Western blot analysis of caspases activities and PARP cleavage in BHV-1-infected cells (2 MOI) for different times (left panel) or for different MOIs for 48 h p.i. (right panel). Data are representative of three separate experiments. β-actin was used as an internal loading control. (**B**) Effect of caspase inhibitors on cell viability. Cells were incubated with 20 μM of each caspase inhibitors for 2 h prior to infection with BHV-1 at 2 MOI for 48 h and cell viability was evaluated by MTT assay. Values are shown as mean ± SD, * *p < 0.05*, ** *p* < 0.01 versus BHV-1 infection alone without inhibitors. (**C**) The inhibitory efficacy of caspase-8 and −9 inhibitors on the activity of caspase-3. Cells were incubated with 20 μM of each caspase inhibitors for 2 h prior to infection with BHV-1 at 2 MOI for 48 h. the activity of caspase-3 was measured by colorimetric assay kit. The relative caspase-3 activity was calculated after the activity of caspase-3 in cells that treated with BHV-1 or inhibitors subtracted the background value. Values are mean ± SD. * *p < 0.05*, ** *p* < 0.01 versus BHV-1 infection alone without inhibitors.

To gain insight into the mechanism underlying BHV-1-induced apoptosis, we investigated the contribution of caspases to BHV-1-induced apoptosis in MDBK cells. The protein levels of caspase-8, -9 and −3 were measured using western blot analysis. Full-length procaspase-8, procaspase-9 and procaspase-3 were cleaved. Their activated form showed a time- and dose-dependent increase upon BHV-1 infection (Figure 2A). PARP, a representative substrate for effector caspases, can be cleaved by caspase-3 [10]. Western blot analysis was performed to detect the PARP and its cleavage fragment. As shown in Figure 2A, the levels of full-length PARP significantly decreased at 24 h p.i., while the levels of cleaved PARP had significantly increased and then further increased at 48 h p.i., however, no cleaved PARP was detected in mock-infected cells. The cleaved PARP level was also appears to increase with MOI of BHV-1 in the infected cells at 48 h p.i. (Figure 2A, right panel).

To further determine the contribution of caspase-8, -9, -3 in apoptosis, we examined the cell viability of BHV-1-infected cells pre-treated with caspase-8, -9 and −3 specific inhibitors z-IETD-FMK, z-LEHD-FMK and Z-DEVD-FMK, respectively. The results show that caspases-8, -9 and −3 specific inhibitors significantly prevent cell death significantly (Figure 2B). To determine the effect of two initiator caspase-8 and −9 in caspase-3 activation, we detected the activity of caspase-3 in the cells pretreated with caspase-8, caspase-9, and both of that inhibitors. The activity of caspase-3 was decreased in cells in the presence of caspase-8 inhibitor or caspase-9 inhibitor. Combining both of the two inhibitors inhibited the activity of caspase-3 more significantly (Figure 2C). Taken together, these results suggest that both extrinsic and intrinsic pathways are involved in caspase-3 activation and BHV-1-induced apoptosis.

### Fas/FasL-mediated apoptotic pathway are involved in BHV-1- induced apoptosis

**Figure 3:**
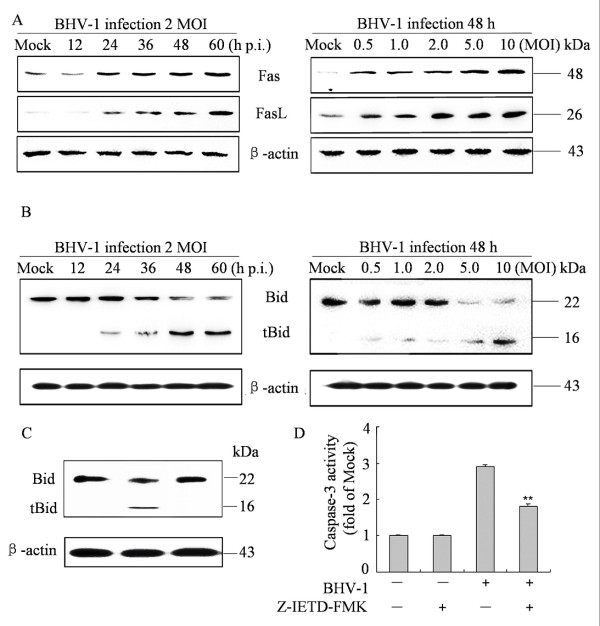
**Effect of BHV-1 infection on FasL, Fas expression and Bid cleavage in MDBK cells.** (**A**) Fas and FasL expression in cells mock-infected or BHV-1-infected (2 MOI) for indicated hours (left panel) or different MOI for 48 h (right panel) were analyzed by Western Blot Analysis. Data are representative of three separate experiments. (**B**) Bid cleavage to tBid in BHV-1-infected cells. Cells were treated as A and subjected to Western Blot Analysis. β-actin was used as internal controls. Data are representative of three separate experiments. (**C**) tBid expression in MDBK cells. Cells were pre-treated with the caspase-8 inhibitor and infected with BHV-1 for 48 h, and then analyzed by Western Blot Analysis. (**D**) Effect of caspase-8 inhibitor on the activity of caspase-9. Cells were pretreated with Z-IETD-FMK for 2 h then infected BHV-1 for 48 h. Caspase-9 activity was measured by a colorimetric assay kit. Data are mean ± SD from three independent experiments. ** *p* < 0.01 versus BHV-1 infection alone.

In some cell types, the extrinsic apoptosis pathway depends on regulating expression of Fas (CD95), which belongs to the tumor necrosis factor (TNF) family. It induces apoptosis by binding its receptor, Fas Ligand (FasL) [11]. Since caspase-8 was activated within the BHV-1-infected MDBK cells, it is possible that BHV-1 infection provokes apoptosis via Fas/FasL pathway. To clarify this, we examined the Fas and FasL levels in BHV-1-infected MDBK cells by western blot analysis. As shown in Figure 3A, FasL and Fas expression increased markedly in a time-and dose-dependent manner.

It is known that activated caspase-8 is able to cleave full-length Bid to tBid, which is important in crosstalk between the death-receptor pathway and the mitochondrial pathway in some cell types [12]. Bid activation results in the destruction of mitochondria integrity and cytochrome c release to cytosol, which further facilitates caspase-9 activation. To test this possibility in BHV-1-induced apoptosis, we determined whether Bid was cleaved upon BHV-1 infection and whether blocking caspase-8 activity could affect Bid cleavage and caspase-9 activation. Western blot analysis revealed that tBid was detected in BHV-1-infected cells but not in mock-infected cells. tBid clearly appeared at 24 h p.i. and maintained a high level in subsequent hours (Figure 3B). Following incubation with z-IETD-FMK, a specific inhibitor of caspase-8, tBid was not detected in BHV-1-infected cells (Figure 3C). However, the activation of caspase-9 was partly blocked in BHV-1-infected cells (Figure 3D). These results suggest that apoptosis signals from the FasL were transmitted to caspase-8 which in turn cleaves Bid and is followed by caspase-9 activation, and that other apoptosis signals also contribute to the activation of caspase-9.

### BHV-1 infection regulates the expression of the Bcl-2 family of proteins and promotes the release of cytochrome c from mitochondria

**Figure 4:**
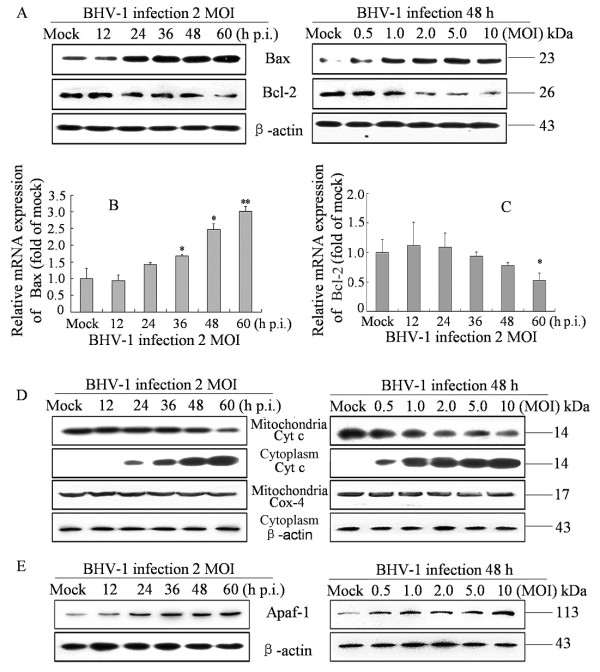
**Effect on release of cytochrome c by regulating Bcl-2 family protein in BHV-1-infected cells.** (**A**) Bcl-2 and Bax protein expression in cells mock-infected or BHV-1-infected (2 MOI) for indicated hours (left panel) or different MOI for 48 h (right panel) were analyzed by Western Blot Analysis. (**B**) qRT-PCR assay for mRNA levels of Bax .Data are mean ± SD from three independent experiments. * *p < 0.05*, ** *p* < 0.01 versus mock infection. (**C**) qRT-PCR assay for mRNA levels of Bcl-2. Data are mean ± SD from three independent experiments. * p < 0.05, ** *p* < 0.01 versus mock infection. (**D**) Cytochrome c release of BHV-1-infected cells. Cells were mock-infected or BHV-1-infected at 2 MOI for different hours, and analyzed by Western Blot Analysis. Cox4 and β-actin were used as internal controls for the mitochondrial fractions and the cytosolic fraction, respectively. Data are representative of three separate experiments. (**E**) Apaf-1 expression in cells mock-infected or BHV-1-infected (2 MOI) for indicated hours (left panel) or different MOI for 48 h (right panel) were analyzed by Western Blot Analysis.

Mitochondria are the central feature of apoptosis regulation. Cytochrome c released from mitochondria to cytoplasm induces subsequent activation of caspase-9. This process is tightly controlled by members of Bcl-2 family. Pro-apoptotic Bcl-2 proteins such as Bax or Bak activated upon apoptosis signals results in outer mitochondrial membrane permeabilization. In contrast, anti-apoptotic Bcl-2 proteins such as Bcl-2 or Bcl-XL can prevent this occurrence [13]. The activation of caspase-9 suggests that BHV-1 infection might disturb the homeostasis of the mitochondria. In our experiment, the levels of Bax and Bcl-2 were detected by western blot analysis and qRT-PCR in BHV-1 infected MDBK cells. Protein levels of Bax exhibited an apparent increase in dose- and time- dependent manner (Figure 4A). Compared with mock infected cells, the mRNA levels of Bax in BHV-1-infected cells were up-regulated as early as 24 h p.i. and reached a peak level at 60 h p.i. (Figure 4B). Conversely, the protein levels of Bcl-2 were down-regulated in a time- and dose- dependent manner in BHV-1 infected cells (Figure 4A). Also, the mRNA levels of Bcl-2 significantly decreased in BHV-1-infected cells at 60 h p.i., (Figure 4C). Next, we observe that cytochrome c released from the mitochondria to cytosol in BHV-1 infected cells was not readily detected in the cytosol fraction of mock-infected cells (Figure 4D). We assume that apoptotic protease-activating factor-1 (Apaf-1), as a central component of apoptosome complex, might be involved in the activation of caspase-9. The results show that Apaf-1 was up-regulated in dose- and time- dependent manner (Figure 4E). Taken together, these results suggest that Bax and Bcl-2 might play roles in regulating the integrity of mitochondria and promoting the release of cytochrome c from mitochondria in BHV-1-infected cells.

### Interaction between virus replication and cell apoptosis

**Figure 5:**
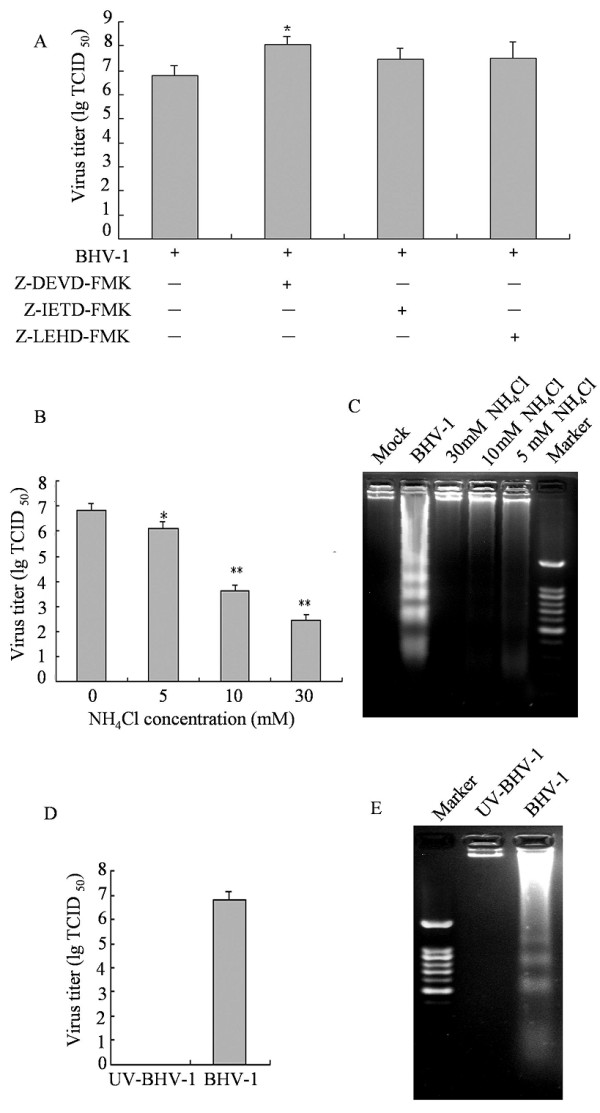
**Inhibitory experiments of BHV-1-induced apoptosis.** (**A**) MDBK cells were pre-treated with inhibitors for 2 h then infected with 2 MOI BHV-1 for 48 h. After incubation, virus were collected and virus titers are shown as lg TCID_50_/ml. (**B**) MDBK cells were pretreated with NH_4_Cl at different concentrations for 2 h before infection and then infected with 2 MOI BHV-1 for 48 h. Cells infected in the same conditions without NH_4_Cl were used as positive controls. Virus titers are shown as lg TCID_50_/ml. (**C**) DNA fragmentation in the presence of NH_4_Cl infected cells was examined by DNA fragmentation assay. (**D**, **E**) BHV-1 was inactivated by exposing to a 30 W UV germicidal light at a distance of 30 cm for 30 min at 4°C. MDBK cells were infected with UV-inactivated virus at 10 MOI for 24 h at 37°C, and than virus titer (**D**) and DNA fragmentation (**E**) were examined.

It has been demonstrated that caspases play an important role in Virus-induced apoptosis and that the pan caspase inhibitor (Z-VAD-FMK) increases virus yield approximately 2 fold in MDBK cells [9]. To test which caspase takes the main role in increasing virus replication, virus progenies released in the presence or absence of the caspase inhibitors were determined by measuring the virus TCID_50_. Figure 5A shows that caspase-3 inhibitor could increase the virus replication dramatically but the caspase-8 caspase-9 inhibitor did not show dramatic effects. Down-stream caspases activation has negative impact on virus life cycle. To determine whether the entry of virion into the cells was necessary for the induction of apoptosis, we used NH_4_Cl to inhibit endosomal acidification, thus impeding the release of virion to the cytoplasm. MDBK cells were pretreated with NH_4_Cl at indicated concentrations for 2 h before infection and then infected with 2 MOI BHV-1 for 48 h. Cells infected in the same conditions without NH_4_Cl were used as positive controls. We observed that NH_4_Cl treatment decreased viral titer and attenuated BHV-1-induced apoptosis (Figure 5B, C). This result suggests that BHV-1-induced apoptosis requires the viral particles to entry MDBK cells.

To further examine whether apoptosis induction by BHV-1 requires virus replication, we used UV treatment to abrogate replication of BHV-1. After BHV-1 was subjected to UV treatment, no viral progeny was detected. Consistently, induced apoptosis disappeared in cells infected with UV-inactivitated BHV-1. The capacity of BHV-1 to induce apoptosis was investigated. When BHV-1 was subjected to UV treatment, a drastic reduction in viral progeny titer was observed (Figure 5D). In cells infected with UV-inactivated BHV-1, apoptosis induction was suppressed when compared to UV-untreated BHV-1 (Figure 5E). Taken together, our results suggests that viral replication is needed for apoptosis induction in BHV-1-infected cells.

## Discussion

Infections with several alphaherpesviruses induce apoptotic cell death in various cell types. Anatid herpesvirus type 1 (AHV-1) infection induces apoptosis in duck embryo fibroblast cells [14]. Herpes simplex virus type 2 (HSV-2) activates the apoptotic process in T cells [15]. For BHV-1, it was reported that the attachment of BHV-1 virions to cells induces apoptotic cell death in PBMC cells [7] and bovine BL-3 (B lymphoma cells) [16]. However, the apoptotic signal pathways in BHV-1-infected cells are not well understood. The stimultaneous activation of caspase-8 and caspase-9 suggests that BHV-1 can trigger the death-receptor pathway and the mitochondrial pathway separately and in parallel. It will be interesting to reveal the upstream signals that trigger these two pathways following BHV-1 infection.

In many cases caspase are required to propagate the signal to commit suicide [17,18]. Many viruses trigger the activation of these caspase cascades, which in turn are responsible for apoptosis induction in infected cells [15]. In BHV-1-infected MDBK cells, the activation of caspase-8, 9 and 3 and cleavage of PARP were observed. Furthermore, incubation of specific caspase inhibitors significantly enhanced the cell viability. These results are consistent with a previous study which showed that PARP is cleaved during BHV-1 infection [9] and suggests that BHV-1-induced apoptosis involves caspase-8 and caspase-9 activation.

Several viruses have been shown to readily activate the caspase-8-associated apoptotic pathway [19]. In the case of BEFV, the induced apoptosis involves increasing the expression of Fas/FasL and activation of caspase-8 [20]. On the other hand, although caspase-8 is often activated through the death-receptor pathway in many systems, the Sendai virus can active apical caspase-8 without involvement of the upstream death receptors during the apoptotic process [21]. Our research provided evidence that BHV-1 induces apoptosis in MDBK cells, involving caspase-8-dependent pathways. This suggests that an extrinsic pathway was involved in the BHV-1-induced apoptosis. The increasing expression of Fas and FasL indicates that caspase-8 activation in BHV-1-infected MDBK cells can be mediated by Fas/FasL signal payhway. However, just how Fas expression is regulated by BHV-1 remains unclear, and further studies are under way to elucidate the mechanism of Fas regulation in BHV-1-infected MDBK cells. Caspase-8 has been shown to activate a mitochondria pathway following the cleavage of Bid, a pro-apoptotic member of Bcl-2 family [22,23]. Cleavage of Bid in BHV-1-infected cells was consistent with such a mechanism. We also propose that the release of cytochrome c from mitochondria and increasing expression of Apaf-1 are critical steps in the BHV-1-initiated apoptosis. These results are consistent with current theory that tBid transfers to the surface of the mitochondria causing cytochrome c release [19]. Thereafter, cytochrome c helps Apaf-1 to recruit and activate caspase-9 preprotein, which in turn activates caspase-3 [24]. Our research provides evidence that BHV-1 infection induced apoptosis occurs through a mitochondria pathway. There are also some other types of virus which induce apoptosis through mitochondria pathway, such as the equine arteritis virus [19] and the dengue virus [25]. Suppressing the activation of caspase-8 and caspase-9 separately degrades the activation of caspase-3 but did not stop it totally, indicating that both the extrinsic and intrinsic pathways contribute to activation of caspase-3 and cleavage of PARP progressively.

Mitochondria-mediated apoptosis is strictly controlled by Bcl-2 family members, which include a number of pro-apoptotic and anti-apoptotic proteins to regulate apoptosis by changing relative levels [26]. In some cases, some viruses either encode proteins homologous to Bcl-2 to inhibit apoptosis or influence Bcl-2 expression to establish a persistent infection. Many viruses inducing apoptosis by modulating expression of Bcl-2 family proteins have been reported [27]. Our results show that the BHV-1 infection up-regulated Bax, down-regulated Bcl-2 expression, and induced the release of cytochrome c, resulting in MDBK cells apoptosis. These results are consistent with the observation in other herpes viruses [28,29], which suggest that modulation of Bcl-2 members is a key step for herpes viruses induction of CPE and eventually apoptosis.

In most cases of virus-induced apoptosis, apoptosis is a result of crosstalk between extrinsic and intrinsic pathways [19,30]. In this pathway, activated caspase-8 cleaves Bid to tBid which, in turn, translocates to mitochondria and initiates the release of cytochrome c into the cytosol activating caspase-9. Our results also provide evidences for the potential cross-talk between extrinsic and intrinsic pathways in BHV-1-induced apoptosis. Blocking caspase-8 activity using z-IETD-FMK, a specific inhibitor of caspase-8, does not completely abrogate caspase-9 activation. This suggests that caspase-9 is activated not only by caspase-8 activation but also by other upstream apoptotic signals. In previous study, P53 appeared to be involved in the BHV-1-induced apoptosis in MDBK cells because the p53 level and promoter activity increased after infection [9].

For some types of viruses, viral replication is required for the induction of apoptosis during infection with these viruses. BHV-1 needs to penetrate MDBK cells in order to trigger cell death, but not to induce host cell intrinsic-recognition mechanism [8]. Pan caspase inhibiter can prevent BHV-1-induced apoptosis and enhance the virus yield [9], in which down-stream caspase (caspase-3) leads a main role. In the meantime, caspase inhibitors increase cells’ viability, and the effect of caspase-3 inhibitor is more significant. We hypothesize that it was because caspase inhibitors prolonged the survival of MDBK cells that more virus was assembled in cells. In other words, apoptosis has a negative impact on the virus life cycle. However, the inhibition of caspases does not affect Canine Coronavirus Type II (CCoV-II) replication in canine fibro sarcoma cells (A-72 cells) [31]. Treatment of MDBK cells with NH_4_Cl or infection with UV-inactivated BHV-1 was seen to abrogate virus apoptosis induction, suggesting that BHV-1-induced apoptosis in MDBK cells depends on viral replication. Although apoptosis occurred in MDBK cells after BHV-1 infection, UV-inactivated virus did not efficiently induce apoptosis. This finding is in contrast to those from previous studied using PBMC or activated CD4^+^ T cells. In general, lymphoid cells are prone to apoptosis, suggesting that inactivated virus can induce apoptosis in cells which easily undergo apoptosis. It is clear that BHV-1 induces cell death in a cell-type-dependent fashion, and this is probably because novel virus-host interactions are important for BHV-1-initiated apoptosis in different cell types. For example, a novel member of the tumor necrosis factor (TNF) NGF receptor family (HVEM) [32] mediates HSV-1 entry into activated T cells. Conversely, entry of HSV-1 into epithelial or other non-lymphoid cells is mediated by an unrelated membrane glycoprotein which resembles the poliovirus receptor [33].

## Conclusions

In summary, our results demonstrate that BHV-1 infection activities caspase-8 through a death receptor pathway by regulating Fas/FasL expression, followed by the involvement of a mitochondria-mediate pathway, which leads to the release of cytochrome c and activation of caspases-9 and −3. Caspases are involved in controlling virus release by inducing infected cells apoptosis. In addition, BHV-1 replication may be necessary for the induction of apoptosis in BHV-1-infected cells, but in the mean time, prolonged cell viability benefits BHV-1 replication.

## Methods

### Antibodies, cells and virus

Monoclonal antibodies against caspase-8, caspase-9, caspase-3, PARP, Fas, FasL, Bid, Bcl-2, Bax, cytochrome c, Apaf-1, Cox4, β-actin were purchased from Santa Cruz Biotechnology, Inc. (Santa Cruz, CA, USA). Horseradish peroxidase (HRP)-conjugated secondary antibody was purchased from Pierce (Pierce, Rockford, IL, US). Madin Darby bovine kidney (MDBK) cells were cultured in Dulbecco’s Modified Eagle Medium (DMEM) (Gibco BRL, Gaithersburg, MD, US) supplemented with 10% heat-inactivated new born bovine serum (Gibco BRL, Gaithersburg, MD, US), 100 IU of penicillin and 100 μg of streptomycin per ml, at 37°C in a 5% CO_2_ atmosphere incubator. The BHV-1 Shaanxi strain, which was isolated and characterized in 2009, was kindly provided by Prof. Jing-Yu Wang, College of Veterinary Medicine, Northwest A&F University [34]. Virus titers were determined by 50% tissue culture infective doses (TCID_50_) as described previously [35].

### Cell viability determination

Cell viability was determined by MTT assay as described previously [36]. Briefly, 5,000 cells were seeded in 96-well plate chambers and then cells were infected with BHV-1 at different MOI (50, 10, 2, 0.4, 0.008, 0.016, and 0.0032 MOI). At every 12 hours post infection (h p.i.), cells were incubated with 3-(4, 5-dimethylthiazol-2-yl)-2, 5-diphenyltetrazolium bromide (MTT, 5 mg/ml) for further 4 h. Then the medium was replaced with DMSO (Dimethyl sulfoxide) to solubilize the formazan crystals. 10 minutes later, absorbance at 570 nm was measured with a microplate reader (BioTek Instruments, Inc., Winooski, US). Appropriate controls were performed incubating cells with DMEM, but without BHV-1. Data are presented as a percentage of the control, and results are the mean ± SD of three independent experiments performed in duplicate.

### Morphological analysis

MDBK cells were grown on slides in 6-well plates and were infected with BHV-1 at 2 MOI. At indicated h p.i. cells were stained with acridine orange (AO, 200 μg /ml) and ethidium bromide (EB, 200 μg /ml) and then washed with phosphate buffer saline (PBS) to remove background staining. After that, slides were covered with cover slips and observed under a fluorescence microscope (Nikon Inc, Japan). The normal cells and early apoptotic cells can be stained by AO to appear bright green fluorescence, while the late apoptotic cells can be stained by EB to appear orange fluorescence.

### DNA fragmentation assay

Mock-infected cells or BHV-1-infected cells were harvested, washed and incubated with lysis buffer (20 mM EDTA, 100 mM Tris, pH 8.0, 0.8% SDS) at room temperature for 1 h. After centrifugation for 10 min at 12000 × *g*, the supernatants were collected and treated with Rnase A (500 μg/ml) for 1 h at 37°C, followed by digestion with proteinase K (500 μg/ml) for 2 h at 55°C. The DNA was extracted using the phenol/chloroform/isoamylol (25:24:1), precipitated with ethanol, dissolved in TE buffer (10 mM Tris, pH 8.0, 1 mM EDTA), and subjected to 2.0% agarose gel electrophoresis for DNA fragmentation analysis.

### DNA content assay

Flow cytometric analysis of apoptosis was performed by analyzing the reduced fluorescence of the DNA binding dye propidium iodide (PI) in the apoptotic nuclei as previously described [37]. Briefly, MDBK cells were seeded in 6-well plastic tissue culture plates and mock infected or infected with BHV-1. Then cells were collected and washed twice with PBS. Following this, cells were fixed and permeabilized by suspension in 70% cold ethanol and incubated overnight at 4°C. Cell pellet was resuspended at room temperature in hypotonic solution consisting of 0.1% sodium citrate pH 6.5, 1% propidium iodide (PI) staining. Cells were analyzed after at least 1 h of incubation at 4 ~ 8°C in the dark with a flow cytometry (Beckman Coulter EPICS ALTRA, Orlando, US). At least 10,000 events were acquired for each sample.

### Measurement of caspases activity

Caspases activities were measured by Caspases (caspase-3 and 9) Colorimetric Assay Kit (BioVision, Inc., Mountain View, California, US), according to manufacturer’s recommendations. Briefly, the cells pellet harvested by centrifugation was incubated for 30 min in chilled cell lysis buffer on ice. The lysate was centrifuged at 4°C and the supernatants were used for caspases activity assay. The supernatants protein content was estimated using BCA Protein Assay Reagent (Pierce, Rockford, IL, US). A total of 200 μg of proteins were incubated for 4 h at 37°C with 200 μM colorimetric substrate. The protease activity was determined by spectrophotometric detection. Absorbance at 405 nm was measured with a microplate spectrophotometer (BioTek Instruments, Inc., Winooski, US).

### Western blot analysis

Cells were harvested and treated with ice-cold RIPA (Radio Immunoprecipitation Assay) lysis buffer with 1 mM phenylmethyl sulfonylfluoride (PMSF). Isolation of mitochondrial and cytosolic proteins was performed using the Mitochondria/cytosol Fractionation Kit (Pierce, Rockford, IL, US). Equivalent amounts of proteins were loaded and electrophoresed on 12% SDS-PAGE and transferred to PVDF membranes (Millipore Corp, Atlanta, GA, US). The membranes were blocked with 5% nonfat dry milk and then incubated with indicated primary antibodies over night at 4°C, followed by HRP-conjugated secondary antibodies. The signal was detected by ECL reagent (Pierce, Rockford, IL, US).

### Quantitative real-time PCR (qRT-PCR) analysis

Total RNA was extracted using TRIzol agent (Invitrogen, California, USA), and 2 μg each RNA sample was reverse-transcribed using First-strand cDNA synthesis kit (Invitrogen, California, USA). The expression of apoptotic regulating genes was quantified using Bio-Rad iQ5 Real Time PCR System by means of a quantitative real-time PCR assay (qRT-PCR). The sets of bovine primer pairs were illustrated in Table 1. Reactions were carried out in 25 μl volume containing 1 × SYBR Premix Ex Taq^TM^ II (Takara, Dalian, China), sense and anti-sense primers (0.4 μM) and target cDNA (4 ng). The cycling conditions were 95°C for 30 s, followed by 40 cycles of 95°C for 5 s, 60°C for 30 s. A negative control was included in each run and the specificity of amplification reaction was checked by melting curve (Tm value) analysis. The relative quantification of gene expression was analyzed by the two-ddCt method [38].

**Table 1:** Sequences of bovine primer pairs used for qRT-PCR

**Gene**	**Forward primer (5**^**′**^**–3**^**′**^**)**	**Reverse primer (5**^**′**^**–3**^**′**^**)**	**Product(bp)**	**Accession no.**
Bax	TCTCCCCGAGAGGTCTTTTT	TGATGGTCCTGATCAACTCG	151	XM_003585205.1
Bcl-2	ATGTGTGTGGAGAGCGTCAA	CTAGGGCCATACAGCTCCAC	146	NM_001166486.1
GAPDHF	ATGCTGGTGCTGAGTATGTG	CTTCTGGGTGGCAGTGAT	293	XM_001252511.3

### Statistical analysis

All data were means ± SD from three independent experiments in triplicate. Results were analyzed by Student’s *t*-test. A *p*-value less than 0.05 was considered to be statistically significant.

## Competing interests

The authors declare that they have no conflicts of interest.

## Authors’ contributions

XX and KZ performed the majority of experiments and involved in manuscript preparation, YH participated in editing of the manuscript. LD, GC and HZ participated part of the experiments. DT conceived of the study, participate in its design and coordination, and revised the manuscript. All authors read and approved the final manuscript.

## Authors’ information

Dr. De-Wen Tong, professor of College of Veterinary Medicine, Northwest A&F University, Vice Dean of College of Veterinary Medicine, Northwest A&F University. Dr. Xin-Gang Xu and Dr. Yong Huang are assosiate professors of College of Veterinary Medicine, Northwest A&F University. Kuan Zhan , Li Ding , Guangda Chen and Honglei Zhang, graduate students of College of Veterinary Medicine, Northwest A&F University.

## References

[B1] Kampa J Stahl K Moreno-López J Chanlun A Aiumlamai S Alenius S BVDV and BHV-1 Infections in Dairy Herds in Northern and Northeastern Thailand Acta Vet Scand 2004 45 181 192 10.1186/1751-0147-45-181 15663078 PMC1820995

[B2] Jones C Chowdhury S A review of the biology of bovine herpesvirus type 1 (BHV-1), its role as a cofactor in the bovine respiratory disease complex and development of improved vaccines Anim Health Res Rev 2007 8 187 205 10.1017/S146625230700134X 18218160

[B3] Prysliak T van der Merwe J Lawman Z Wilson D Townsend H Perez-Casal J Van Drunen Littel-van den Hurk S Respiratory disease caused by Mycoplasma bovis is enhanced by exposure to bovine herpes virus 1 (BHV-1) but not to bovine viral diarrhea virus (BVDV) type 2 Can Vet Journal 2011 52 1195 1202 PMC3196011 22547839

[B4] Winkler M Doster A Jones C Bovine herpesvirus 1 can infect CD4+ T lymphocytes and induce programmed cell death during acute infection of cattle J Virol 1999 73 8657 8668 10482619 10.1128/jvi.73.10.8657-8668.1999 PMC112886

[B5] Fulda S Debatin K Extrinsic versus intrinsic apoptosis pathways in anticancer chemotherapy Oncogene 2006 25 4798 4811 10.1038/sj.onc.1209608 16892092

[B6] Clarke P Tyler KL Apoptosis in animal models of virus-induced disease Nat Rev Microbiol 2009 7 144 155 10.1038/nrmicro2071 19148180 PMC2772826

[B7] Hanon E Lambot M Hoornaert S Lyaku J Pastoret P Bovine herpesvirus 1-induced apoptosis: phenotypic characterization of susceptible peripheral blood mononuclear cells Arch Virol 1998 143 441 452 10.1007/s007050050301 9572546

[B8] Hanon E Meyer G Vanderplasschen A Dessy-Doizé C Thiry E Pastoret PP Attachment but not penetration of bovine herpesvirus 1 is necessary to induce apoptosis in target cells J Virol 1998 72 7638 7641 9696867 10.1128/jvi.72.9.7638-7641.1998 PMC110026

[B9] Devireddy LR Jones CJ Activation of caspases and p53 by bovine herpesvirus 1 infection results in programmed cell death and efficient virus release J Virol 1999 73 3778 3788 10196272 10.1128/jvi.73.5.3778-3788.1999 PMC104155

[B10] Herceg Z Wang ZQ Functions of poly (ADP-ribose) polymerase (PARP) in DNA repair, genomic integrity and cell death Mutat Res Fundam Mol Mech Mutagen 2001 477 97 110 10.1016/S0027-5107(01)00111-7 11376691

[B11] Scaffidi C Fulda S Srinivasan A Friesen C Li F Tomaselli KJ Debatin KM Krammer PH Peter ME Two CD95 (APO-1/Fas) signaling pathways EMBO J 1998 17 1675 1687 10.1093/emboj/17.6.1675 9501089 PMC1170515

[B12] Sprick MR Walczak H The interplay between the Bcl-2 family and death receptor-mediated apoptosis Biochimica et Biophysica Acta (BBA)-Molecular Cell Research 2004 1644 125 132 10.1016/j.bbamcr.2003.11.002 14996497

[B13] Shimizu S Narita M Tsujimoto Y Bcl-2 family proteins regulate the release of apoptogenic cytochrome c by the mitochondrial channel VDAC Nature 1999 399 483 487 10.1038/20959 10365962

[B14] Yufei G Chanjuan S Anchun C Mingshu W Na Z Shun C Yi Z Anatid herpesvirus 1 CH virulent strain induces syncytium and apoptosis in duck embryo fibroblast cultures Vet Microbiol 2009 138 258 265 10.1016/j.vetmic.2009.04.006 19410389 PMC7126888

[B15] Oever MJV Han JY Caspase 9 is essential for herpes simplex virus type 2-induced apoptosis in T cells J Virol 2010 84 3116 3120 10.1128/JVI.01726-09 20071584 PMC2826057

[B16] Geiser V Rose S Jones C Bovine herpesvirus type 1 induces cell death by a cell-type-dependent fashion Microb Pathog 2008 44 459 466 10.1016/j.micpath.2007.10.014 18222625 PMC2459245

[B17] Rodríguez-Berriguete G Galvis L Fraile B de Bethencourt FR Martínez-Onsurbe P Olmedilla G Paniagua R Royuela M Immunoreactivity to caspase-3, caspase-7, caspase-8, and caspase-9 forms is frequently lost in human prostate tumors Hum Pathol 2012 43 229 237 10.1016/j.humpath.2011.04.024 21802116

[B18] Vaisid T Barnoy S Kosower NS Calpain activates caspase-8 in neuron-like differentiated PC12 cells via the amyloid-β-peptide and CD95 pathways Int J Biochem Cell Biol 2009 41 2450 2458 10.1016/j.biocel.2009.07.010 19646546

[B19] St-Louis MC Archambault D The equine arteritis virus induces apoptosis via caspase-8 and mitochondria-dependent caspase-9 activation Virology 2007 367 147 155 10.1016/j.virol.2007.05.023 17583760

[B20] Lin CH Shih WL Lin FL Hsieh YC Kuo YR Liao MH Liu HJ Bovine ephemeral fever virus-induced apoptosis requires virus gene expression and activation of Fas and mitochondrial signaling pathway Apoptosis 2009 14 864 877 10.1007/s10495-009-0371-5 19521777

[B21] Bitzer M Prinz F Bauer M Spiegel M Neubert WJ Gregor M Schulze-Osthoff K Lauer U Sendai virus infection induces apoptosis through activation of caspase-8 (FLICE) and caspase-3 (CPP32) J Virol 1999 73 702 708 9847376 10.1128/jvi.73.1.702-708.1999 PMC103877

[B22] Kantari C Walczak H Caspase-8 and bid: caught in the act between death receptors and mitochondria Biochimica et Biophysica Acta (BBA)-Molecular Cell Research 2011 1813 558 563 10.1016/j.bbamcr.2011.01.026 21295084

[B23] Kim BM Chung HW Hypoxia/reoxygenation induces apoptosis through a ROS-mediated caspase-8/Bid/Bax pathway in human lymphocytes Biochem Biophys Res Commun 2007 363 745 750 10.1016/j.bbrc.2007.09.024 17904098

[B24] Li P Nijhawan D Budihardjo I Srinivasula SM Ahmad M Alnemri ES Wang X Cytochrome c and dATP-dependent formation of Apaf-1/caspase-9 complex initiates an apoptotic protease cascade Cell 1997 91 479 489 10.1016/S0092-8674(00)80434-1 9390557

[B25] Su HL Lin YL Yu HP Tsao CH Chen LK Liu YT Liao CL The effect of human bcl-2 and bcl-X genes on dengue virus-induced apoptosis in cultured cells Virology 2001 282 141 153 10.1006/viro.2000.0820 11259197

[B26] Lindsay J Esposti MD Gilmore AP Bcl-2 proteins and mitochondria-specificity in membrane targeting for death Biochimica et Biophysica Acta (BBA)-Molecular Cell Research 2011 1813 532 539 10.1016/j.bbamcr.2010.10.017 21056595

[B27] Gangappa S Van Dyk LF Jewett TJ Speck SH Virgin HW Identification of the in vivo role of a viral bcl-2 J Exp Med 2002 195 931 940 10.1084/jem.20011825 11927636 PMC2193719

[B28] Li L Chi J Zhou F Guo D Wang F Liu G Zhang C Yao K Human herpesvirus 6A induces apoptosis of HSB-2 cells via a mitochondrion-related caspase pathway Journal of Biomedical Research 2010 24 444 451 10.1016/S1674-8301(10)60059-0 PMC3596692 23554661

[B29] Li L Chi J Zhou F Guo D Wang F Liu G Zhang C Yao K Reactive oxygen species and p38 MAPK regulate Bax translocation and calcium redistribution in salubrinal-induced apoptosis of EBV-transformed B cells Journal of Biomedical Research 2011 313 235 248 10.1016/j.canlet.2011.09.011 22056078

[B30] De Martino L Marféb G Longo M Fiorito F Montagnaro S Iovane V Decaro N Pagnini U Bid cleavage, cytochrome c release and caspase activation in canine coronavirus-induced apoptosis Vet Microbiol 2010 141 36 45 10.1016/j.vetmic.2009.09.001 19781871 PMC7117139

[B31] Marfè G Tafani M Fiorito F Pagnini U Iovane G De Martino L Involvement of FOXO Transcription Factors, TRAIL-FasL/Fas, and Sirtuin Proteins Family in Canine Coronavirus Type II-Induced Apoptosis PLoS One 2011 6 e27313 10.1371/journal.pone.0027313 22087287 PMC3210785

[B32] Montgomery RI Warner MS Lum BJ Spear PG Herpes simplex virus-1 entry into cells mediated by a novel member of the TNF/NGF receptor family Cell 1996 87 427 436 10.1016/S0092-8674(00)81363-X 8898196

[B33] Geraghty RJ Krummenacher C Cohen GH Eisenberg RJ Spear PG Entry of alphaherpesviruses mediated by poliovirus receptor-related protein 1 and poliovirus receptor Science 1998 280 1618 1620 10.1126/science.280.5369.1618 9616127

[B34] Li H Wang J Liu H Zhang S Isolation and identification of infectious bovine rhinotracheitis virus Journal of Northwest A & F University(Natural Science Edition) 2010 38 19 22

[B35] LaBarre DD Lowy RJ Improvements in methods for calculating virus titer estimates from TCID50 and plaque assays J Virol Methods 2001 96 107 126 10.1016/S0166-0934(01)00316-0 11445142

[B36] Li Z Xu X Huang Y Ding L Wang Z Yu G Xu D Li W Tong D Swainsonine activates Mitochondria-mediated apoptotic pathway in human lung cancer A549 cells and retards the growth of lung cancer xenografts Int J Biol Sci 2012 8 394 405 22393311 10.7150/ijbs.3882 PMC3291856

[B37] Lin PY Lee JW Liao MH Hsu HY Chiu SJ Liu HJ Shih WL Modulation of p53 by mitogen-activated protein kinase pathways and protein kinase C [delta] during avian reovirus S1133-induced apoptosis Virology 2009 385 323 334 10.1016/j.virol.2008.12.028 19168198

[B38] Livak KJ Schmittgen TD Analysis of relative gene expression data using real-time quantitative PCR and the 2-[Delta][Delta] CT method Methods 2001 25 402 408 10.1006/meth.2001.1262 11846609

